# Measuring delivery and impact in community-based health promotion initiatives: development and overview of the Healthway Evaluation Framework

**DOI:** 10.3389/fpubh.2025.1676965

**Published:** 2025-12-02

**Authors:** Aaron Simpson, Ben Jackson, Michael Rosenberg, Claire Willis

**Affiliations:** 1 School of Human Sciences (Exercise and Sport Science), The University of Western Australia, Perth, WA, Australia; 2 The Kids Research Institute Australia, Perth, WA, Australia

**Keywords:** evaluation framework design, health promotion evaluation, public health, community health, capacity building

## Abstract

**Background:**

Robust evaluation is critical for understanding and enhancing the impact of health promotion initiatives. However, many community-based organisations face challenges in planning and conducting evaluation due to limitations in knowledge, resources, and the applicability of existing evaluation frameworks. The Healthway Evaluation Framework, and its accompanying practical Measurement Toolkit, was designed to support evaluation planning, implementation, and reporting across diverse health promotion programs and settings.

**Methods and results:**

Drawing on an evidence review and extensive consultation with community organisations and health promotion funders, the Framework consists of four pillars: (i) Activity; (ii) Knowledge, Attitude, Behaviour; (iii) Organisational; and (iv) Sustainability. Each pillar includes clearly defined elements to guide the evaluation of both delivery processes and health and organisational outcomes. A Measurement Toolkit accompanies the Evaluation Framework, providing practical guidance for data collection in health promotion evaluation.

**Conclusion:**

The Healthway Evaluation Framework provides a practical and adaptable solution for advancing evaluation practice, enhancing the consistency of reporting, and strengthening the evidence base for health promotion.

## 1 Introduction

Health promotion refers to “the process of enabling people to increase control over, and to improve their health” (1, p. 4). Health promotion initiatives play an important role in improving population-level health and wellbeing, and achieving global health objectives (2). Aimed at the risk factors for noncommunicable (e.g., smoking, physical inactivity, poor nutrition) or communicable (e.g., COVID-19, Human Immunodeficiency Virus) diseases, health promotion messages and initiatives are often delivered by government departments, or by non-government organisations whose specific purpose is health promotion (3). Substantial investments in health promotion are made by many countries’ governments [e.g., Australia; (4)], and significant returns on investment are typically observed, with the long-term savings of health promotion investments outweighing the short-term costs (5). To maximize the reach and effectiveness of health promotion initiatives, settings-based approaches [i.e., where people live, work, and play; see (6)] can be harnessed through schools (7), workplaces (8), community and arts organisations [e.g., (9)], and sports associations or clubs (10). Delivering health promotion initiatives through these settings offers the potential for large-scale reach in the community, as well as embedding support for healthy behaviours within the environments where people spend significant portions of their time (11, 12).

The robust evaluation of health promotion initiatives is crucial for their success, scalability, and sustainability. Summarized succinctly in the cover text of Rootman et al.’s book (13), “health promotion initiatives need effective evaluation to realize their potential: both to prove their value as investments and to increase their effectiveness in achieving their aims.” Evaluation is essential in relation to understanding the *process* of implementing an initiative or program, as well as the *impact* (or *effect*) it has on outcomes of interest (14). Comprehensive evaluation allows for understanding of whether an initiative is effective in achieving intended health outcomes, whether and how it is feasible and sustainable, and how it can be modified and scaled (15). This approach to evaluation is consistent with ‘hybrid’ effectiveness-implementation trial designs that prioritize the translation of research into practice [see (16)].

Evaluation frameworks or models are designed to provide a ‘scaffold’ for researchers and community organisations by bringing structure to their chosen evaluation objectives, approaches, and methods (17). Many of these frameworks encompass outcome- and process-related features [as an example of a widely used framework, see RE-AIM; (18)]. Some frameworks explicitly guide evaluation planning and methods [e.g., the Process Evaluation Plan; (19)], whereas others include considerations for evaluation within broader guidelines around the development of programs and interventions [e.g., Intervention Mapping; (20, 21)]. The existence of these well-established frameworks *should* foster insightful, robust, and consistent evaluation practices—yet, evaluation within community-based health promotion initiatives is often poorly conducted or not done at all (21). Larger projects with more funding are more likely to have higher evaluation quality (22); however, there are multiple other contributors to inadequate (or a complete absence of) evaluation including time, financial, capacity, and knowledge challenges within the community organisations that deliver health promotion initiatives (23, 24). Additionally, outside of meeting funders’ reporting obligations, conducting comprehensive evaluations requires program deliverers to place value on evaluation and strategically plan health promotion programs with evaluation in mind (25). These underlying challenges to high quality evaluation are exacerbated in low resource settings (26). Despite the existence of frameworks to support evaluation, the absence or limited quality of evaluation efforts in health promotion initiatives underscores the need to better support community organisations to conduct such work, and to better understand what makes evaluation frameworks useful (24).

Several recommendations have been offered to improve evaluation framework design and strengthen health promotion evaluation efforts. First, evaluation frameworks should contain guidance on methods and approaches that are adaptable to the specific context and health target (27). This guidance includes encouraging both quantitative and qualitative methods, tailored to gathering evidence of both process (i.e., delivery) and impact (i.e., outcome) evaluation (28). Close consideration of the contextual circumstances of health promotion initiatives is also crucial (29). Second, the design of evaluation frameworks needs to be more closely informed by stakeholders at all levels (30)—that is, by those who are likely to be the users and beneficiaries of the frameworks and evidence they generate, the end-recipients of health promotion initiatives, and those who administer funding and set policy. Third, evaluation frameworks need to consist of clearly defined outcomes, objectives, and processes—existing frameworks often feature broad guidance that diminishes their practicality and applicability for on-the-ground health promotion practitioners (17). Additionally, it is recognized that the inclusion of measurement tools that sit alongside evaluation frameworks may reduce barriers to their usage and support capacity for implementing evaluation (27). Finally, to inform truly comprehensive evaluation efforts, increased attention is needed toward capturing sustainability evidence, as well as broader components of an initiative or program beyond their impact on specific health status indicators (17). Such sustainability evidence may be captured, for example, through measuring partnerships, organisational capacity changes, and policy and structure reforms.

Healthway (the *Western Australian Health Promotion Foundation*), established in 1991 (see www.healthway.wa.gov.au), is a State Government agency in Western Australia dedicated to health promotion programs and research. Through advocacy, partnerships, and research and evaluation, Healthway’s mandate is to drive positive change in healthy eating, physical activity, smoking and e-cigarette use, alcohol use, and mental wellbeing [see (31)]. The initiative that we introduce and report on in this paper—the *Healthway Evaluation Framework*—was designed in consultation with stakeholders from Healthway, Western Australian community organisations, and health promotion funding recipients to (a) address some of the shortcomings of existing evaluation frameworks (e.g., lack of practicality or measurement guidance, adaptability), (b) improve consistency in health promotion project planning and delivery, (c) improve consistency and quality in evaluation planning and reporting, and (d) build evaluation capacity among community organisations and their partners. Below, we introduce the *Healthway Evaluation Framework*, outline its development, detail its potential for supporting evaluation and project design efforts (not just in relation to Healthway’s funded projects, but for all health promotion evaluation efforts), and highlight the opportunity for its use in augmenting the impact of health promotion initiatives.

## 2 The Healthway Evaluation Framework and Measurement Toolkit

**Figure 1:**
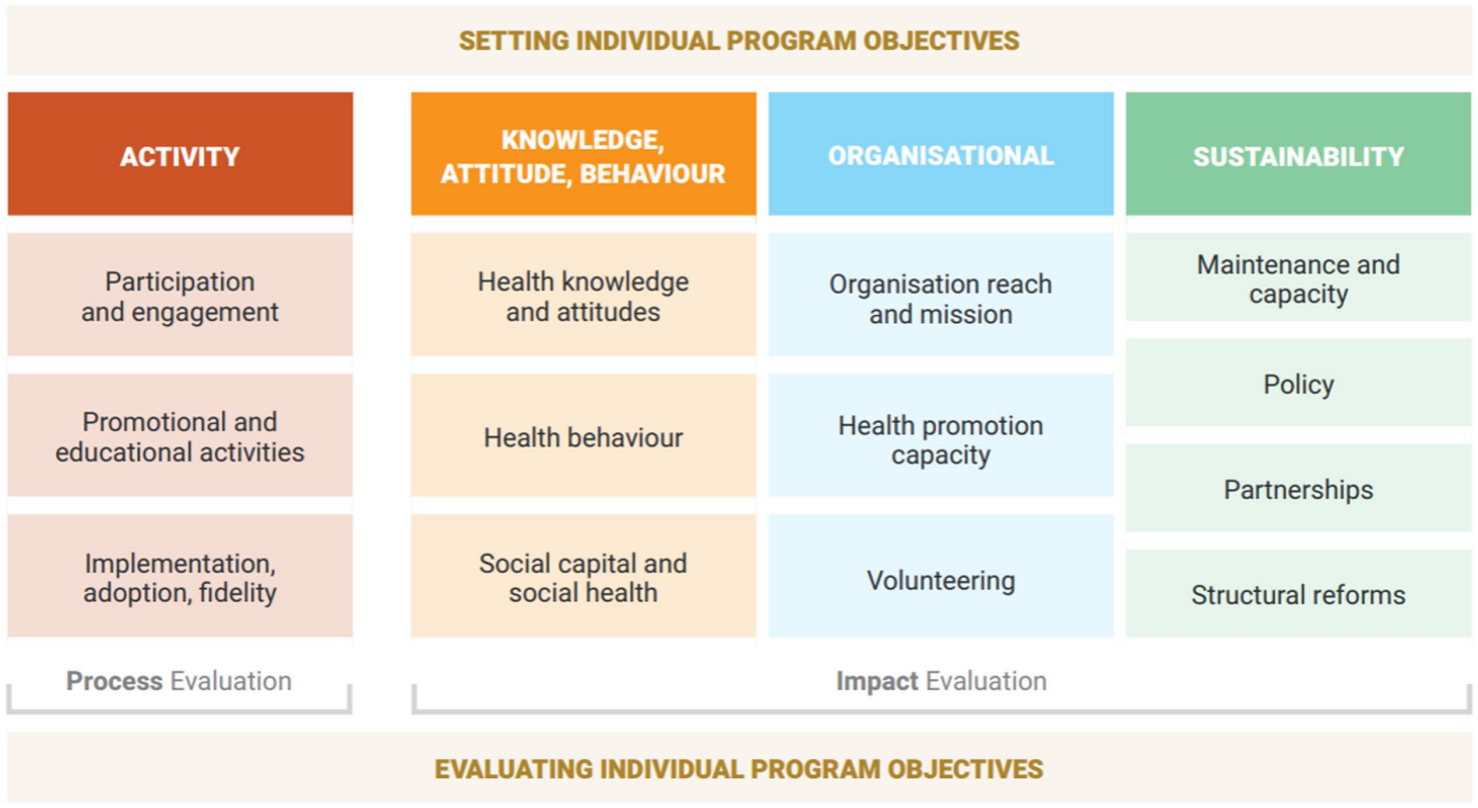
The Healthway Evaluation Framework.

The Healthway Evaluation Framework (see Figure 1 and Supplementary materials) was designed in 2023–2024 and launched in late 2024, with the aim of supporting rigorous evaluation activity for diverse health promotion projects across sports, arts, and other community settings. Although the Framework was developed with the context of Healthway and health promotion in Western Australia in mind, it has applicability to the broader field of health promotion practice and research, and offers a practical, evidence-based approach to scaffolding evaluation efforts globally. The Framework was developed to build evaluation capacity among deliverers of health promotion initiatives and facilitate clear project objectives, evaluation strategy planning, and delivery of evaluation activities that align with the objectives of health promotion organisations and funders. Importantly, the Framework was intended to embed best-practice evidence for process and impact evaluation into a clear and accessible guide for community organisations to understand and implement. Alongside the Evaluation Framework, and guided by recommendations for implementation outlined above, we also developed an accompanying Measurement Toolkit providing organisations with an inventory of measurement items and guidance on how to collect high-quality evidence for their health promotion initiatives.

### 2.1 Development of the framework

The development of the Framework included two stages, involving (i) a review of the literature to identify existing frameworks and current gaps, and (ii) consultation with Healthway and funded agencies and organisations. In our review of the literature, we searched scientific databases for reviews of health promotion research, and for frameworks commonly used to guide health promotion evaluation. Findings and recommendations for health promotion evaluations were collated from identified literature (17, 22–24, 35, 36), and informed early development of the framework. For example, the inclusion of the organisational and Sustainability pillars in the Framework aligns with gaps and recommendations identified in these reviews [e.g., (17)].

The Framework was also mapped against the action areas of the Ottawa Charter for Health Promotion (32) to ensure relevance for global efforts in health promotion. In particular, the Knowledge, Attitude, Behaviour pillar captures the pursuit of *developing personal skills*, the Organisational pillar addresses the need for capacity building in *strengthening community actions* and *creating supportive environments*, and the Sustainability pillar encompasses *building healthy public policy* and *moving into the future*. Additionally, established evaluation and implementation frameworks were studied to ensure comprehensive coverage of elements critical to health promotion evaluation. For example, the RE-AIM framework [see (18)] is a prominent model outlining the importance of assessing reach, efficacy, adoption, implementation, and maintenance during program evaluations. These five elements feature throughout the Healthway Evaluation Framework—reach, adoption, and implementation are included within the Activity pillar; efficacy forms part of the Knowledge, Attitude, Behaviour and Organisational Pillars; and maintenance is captured within the Sustainability Pillar. Other prominent frameworks from the implementation science [such as the implementation outcomes framework outlined by Proctor et al. (33)] and health promotion [such as the PRECEDE-PROCEED model (37)] literatures also informed the development of the Healthway Evaluation Framework. Finally, we consulted evaluation activity [internal documents and scientific publications, e.g., (38)] previously conducted for (and by) Healthway over the past 20 years.

In addition to the literature review, we consulted stakeholders within Healthway and their funded organisations through a series of targeted conversations. Stakeholders from 20 organisations contributed to the consultation process—conversations centered on organisational needs and capacity (e.g., how an evaluation framework could be designed to best support their organisations’ needs), experiences with evaluation (e.g., what worked and what did not), and perspectives on what the focus should be for evaluation of Healthway-funded projects going forward. Following the initial round of consultation, follow-up conversations were held with Healthway (including the Board, executive team, and project officers) over the course of the development process to ensure the Framework aligned with Healthway’s activities and strategic objectives.

### 2.2 Components of the Healthway Evaluation Framework

The Framework includes four ‘pillars’ that represent key domains of health promotion evaluation: (i) Activity; (ii) Knowledge, Attitude, and Behaviour; (iii) Organisational capacity; and (iv) Sustainability. The Activity pillar of the Framework addresses the process of implementing a health promotion initiative, and the other three pillars reflect the evaluation of its impact. Each pillar includes specific elements that, taken together, provide a comprehensive scope of potential delivery and impact outcomes that are important to capture within project planning, project delivery, and evaluation.

The *Activity* pillar of the Framework captures aspects of health promotion project delivery. Often, delivery organisations as a matter of course collect information on aspects of project delivery within their standard reporting and documentation (e.g., event attendance, participant demographics). The Framework provides a structure on how to scaffold the collection of relevant evidence across three discrete elements within the activity pillar: (i) Participation and Engagement; (ii) Promotional and Educational Activities; and (iii) Implementation, Adoption, and Fidelity. Data captured here include, for example, participation among key target populations, the type of activity, and/or whether project activities were delivered as planned. It is important to note that when collecting Activity data—and all evaluation data—efforts should be made to adhere to ethical data management principles (e.g., anonymity, confidentiality, data storage security). See Table 1 for an overview of these elements and an illustration of their measurement.

**Table 1:** The activity pillar.

Element	Description	Measurement
Participation & engagement	The number and type of people and groups that are participating in an activity—including those within the recipient organisation, those delivering activities on behalf of the organisation, and the recipients or consumers of those activities.	Typically measured through counts, website clicks, and/or registrations. Demographic survey items can support tracking of engagement with a funder’s or organisation’s priority populations. Data collected may also include for how long people participated in an activity, or their satisfaction with the activity (e.g., a simple scale of emojis that participants click as they leave).
Promotional & educational activities	A comprehensive audit / record of the various activities, events, strategies, and initiatives provided within the project.	Promotional and educational activities are often collected by organisations as a matter of course—for example, the number of workshops delivered, the presence of signage at an event, and/or the areas (e.g., metropolitan, rural) in which activities were carried out.
Implementation, adoption, and fidelity	All remaining ‘delivery’ considerations informing how the project / activities were implemented and offering insight into reach, scalability, and effectiveness.	Outcomes measured in this element align with Proctor et al.’s (33) implementation outcomes framework. Survey items specifically targeting acceptability, adoption, appropriateness, cost, feasibility, fidelity, penetration, and sustainability can be used here—as well as qualitative data collection (e.g., interviews) to understand what changes can be made and how they would support ongoing implementation, scale-up, and/or scale-out.

The *Knowledge, Attitude, Behaviour* pillar of the Framework focuses on the health behaviours and outcomes associated with health promotion initiatives. Health areas and objectives that fall within health promotion initiatives and projects often vary—the elements within this pillar are designed to provide specific guidance on capturing health impact for any core health promotion objective. Additionally, this pillar was designed to allow for evaluation activity focused on different target populations that might experience changes in knowledge, attitude, behaviour—and not just the end ‘beneficiaries’ of a health promotion initiative. The Knowledge, Attitude, Behaviour pillar can be assessed across three groups—including, for example, staff within the administering health promotion organisation, intermediaries involved in the delivery of a project on the organisation’s behalf (e.g., volunteers, sport coaches), and the community recipients or intended ‘targets’ of the health promotion initiative. Importantly, elements of this pillar may be assessed differently depending on the constraints of the project. In some instances, organisations may be able to collect data at multiple timepoints to understand changes in knowledge, attitudes, behaviours. In other circumstances, it may only be possible to collect data at a single timepoint—in this case, questions need to have the notion of *change* built into them (e.g., how much has this project changed your knowledge of healthy eating?). Additionally, some elements, such as understanding of a health message, may be better assessed with open-ended questioning. This pillar contains three core elements: (i) Health Knowledge and Attitude; (ii) Health Behaviour; and (iii) Social Capital and Social Health. See Table 2 for an overview of these elements and an illustration of their measurement.

**Table 2:** The Knowledge, Attitude, Behaviour pillar.

Element	Description	Measurement
Health knowledge & attitudes	Awareness of a health message (or issue), attitudes toward a health message (or issue), knowledge and confidence regarding that message (or issue), and a person’s ability to understand and apply health messages or strategies.	When health messages are promoted, cognitive impact can be measured through survey items addressing awareness, comprehension, and acceptance of the health message, and intentions, confidence, and actions related to that message / behaviour. Questions can also capture health literacy broadly, or specific to a health target—for example, “*Following participation in the activity, to what extent do you feel your understanding of mental health has changed?*.”
Health behaviour	Changes in (or status of) health indicators that are relevant to project aims, including physical and mental health (and wellbeing) indicators, and specific health-promoting or health-harming behaviours (e.g., sleep, physical activity, smoking, alcohol use).	General health status can be assessed through single-item measures, such as “*in general, would you say your physical health is…?*” on a 1–5 scale from poor to excellent [see (34)]. However, detail on specific health behaviours (frequency, duration, type of relevant behaviours) should be collected where possible. Additionally, wearable data or observational tools can be used as other methods of measuring health behaviours.
Social capital & social health	The social norms, networks, cohesion, trust, and/or supports that underpin cooperation, provide resources and development opportunities, and add value to an individual’s experience.	Survey items can measure the number of people someone participates in a health promotion activity with, and their trust in and connection to those people. Additionally, data collection may pertain to more general measures of social engagement, connection to community, and social support networks. Qualitative data collection is particularly useful here for understanding the nature of a participant’s social capital, and how or why a project has supported their social connections.

Although the guidance in this table predominantly focuses on the measurement of primary, individual-level Knowledge, Attitude, Behaviour data, there may also be opportunities to assess these elements through population-level or secondary data sources. The suitability of such datasets depends on the context, behaviours, and population of interest. However, for many health promotion projects, primary data collection as outlined in the table will be most appropriate.

The *organisational* pillar addresses outcomes of an initiative for the health promotion organisation itself, including in relation to its reach, mission, capacity, and people. This pillar was developed in recognition of the need to help organisations better understand the impact that health promotion initiatives have on *their own* capacity to reach their target populations, to align their health promotion efforts with their organisational purpose, and to support people involved in the project delivery. The organisational pillar consists of three elements: (i) organisations Reach and Mission; (ii) Health Promotion Capacity; and (iii) Volunteering. See Table 3 for an overview of these elements and an illustration of their measurement.

**Table 3:** The Organisational pillar.

Element	Description	Measurement
Organisation reach & mission	Whether and how the funded project and activities supported the organisation’s reach and mission.	Interviews with organisation staff allow for in-depth exploration of how a project advanced an organisation’s mission, in what ways, and how the mission could be expanded in the future. For measuring reach, in addition to counting participants and demographic data collection, survey items for organisation staff may be framed to understand whether the organisation’s reach expanded following an event (e.g., receiving funding), and the degree to which priority populations were reached as intended.
Health promotion capacity	The short-, medium-, and longer-term benefits through lasting change to the organisation’s capacity for designing and delivering health promotion and evidence-building activities.	Capturing change in organisational capacity can relate to health areas (e.g., “*change in your organisation’s capacity to deliver initiatives to improve physical activity*”) and aspects of delivery (e.g., “*change in your organisation’s staffing levels*”). Questions may also be targeted to individual aspects of capacity, such as self-efficacy, confidence, and knowledge, among key staff members. Documentation of strategic plans, budget allocations, organisational structure, and activity data all also contribute to demonstrating change in capacity.
Volunteering	The benefits and costs to organisations regarding volunteers (and to volunteers themselves).	Survey items may be asked of an organisation (e.g., the number of volunteers involved in a project, change in volunteer counts, and importance of volunteers in delivering activities), as well as of volunteers (e.g., burden of volunteering and the challenges involved, positive effects related to volunteering, or connection to the organisations). Interviews or focus groups with volunteers will provide detailed information about experiences as a volunteer and recommendations for volunteering in the project in the future.

The *Sustainability* pillar centers on the elements that support (or hinder) the long-term viability and scale of delivering a health promotion project—particularly, beyond an initial funding period. Planning for (and evaluating) sustainability should be a core consideration of all health promotion initiatives (17). Guidance within this pillar supports assessment of the strategies, partnerships, and policies that arise from and that meaningfully influence ongoing project delivery and potential changes to the organisation’s overall operation. Four elements sit within the Sustainability pillar: (i) Maintenance and Capacity; (ii) Policy; (iii) Partnerships; and (iv) Structural Reforms. See Table 4 for an overview of these elements and an illustration of their measurement.

**Table 4:** The sustainability pillar.

Element	Description	Measurement
Maintenance & capacity	An organisation’s overall capacity to maintain the funded project or initiative beyond its initial funding window or lifespan.	The support, policies, strategies, and staffing to continue the project in an ongoing capacity can be measured quantitatively (to assess the presence of the above) and qualitatively (to assess the nature of the above). Understanding barriers and facilitators to long-term project sustainability, and recording changes in policies and strategies to address these barriers, is also important.
Policy	The development, implementation, and sustainability of any (internal or external) policy change resulting directly or indirectly (e.g., through a change in organisational capacity) from the project.	Measured directly through changes in policies (e.g., updates to policies enacted at an organisation’s activities, such as only serving water and non-sugary drinks at events), as well as indirectly through qualitative assessment of perceptions of policy change. The implementation of policies can be collected from organisation staff, as well as recipients (e.g., insight into whether recipients believed intended policies had been implemented).
Partnerships	The quality and nature of partnerships with external organisations that are necessary to support program sustainability and (if applicable) scalability.	Qualitative data collection provides insight into the nature of partnerships (e.g., why certain partnerships are central to a project, and how the organisations engaged with partners). The presence of, and change in, partnerships to deliver a health promotion project can be collected quantitatively through record keeping and survey items.
Structural reforms	Physical, structural, and organisational changes made within the organisations (or the organisation’s reach) that contribute to project sustainability.	Survey items capturing organisations staff perspectives on reforms to their organisation’s budget or strategic plan, physical sites or locations, and organisational processes. Specific insight on how effective these reforms have been, and how sustainable they are in the medium- and long-term, can be gathered through targeted interview questions or scale-based survey questions.

In summary, the Healthway Evaluation Framework was designed to address the ‘gaps’ in existing evaluation frameworks—it is adaptable, informed by stakeholders, provides clearly defined guidance for measurement, and captures a comprehensive range of evaluation features. The flexibility of the Framework allows organisations (or evaluators) to cover only the pillars and elements relevant to their project’s objectives and activities. And, although aligning evaluation with the Framework early in the project planning stage is most beneficial and will promote awareness of possible delivery methods and the impact that might be possible, the Framework can be implemented at any stage of the health promotion project implementation process. Uptake in aligning evaluation work with the Framework will facilitate clearer communication, planning, and reporting—giving a ‘common language’ that can be used in funding submissions, reporting, and acquittals. Finally, the Framework is designed to be useful across settings, contexts, and health behaviours (e.g., physical activity, healthy eating, smoking, alcohol, mental health and wellbeing promotion), and relevant to any organisations focused on health promotion regardless of the delivery setting (e.g., sport, arts, community outreach).

### 2.3 The Healthway Measurement Toolkit

The Healthway Measurement Toolkit was developed alongside the Evaluation Framework as part of the core objective of making the Framework practical and easy to implement for health promotion organisations [aligning with researchers’ calls for practicality; e.g., (17)]. Additionally, the Toolkit promotes the standardization of questions across multiple projects, supporting efforts to aggregate data and understand collective impact of funded projects. The Toolkit includes a detailed repository of measurement items for collecting data—against all elements within the Framework—for evaluation and reporting of health promotion initiatives. We aligned the Toolkit with the Healthway Evaluation Framework, streamlining the evaluation planning process for organisations and improving the consistency with which they can report process and impact outcomes. The Toolkit contains a combination of newly-developed survey items, items adapted from previous Healthway evaluation activity, and items adapted from published sources and measurement tools (with citations to original sources). And, in line with recommendations in the literature (see above), we also included guidance on when and how to collect quantitative and qualitative data, and recommendations for community involvement in the design and evaluation of health promotion initiatives. In addition to supporting the use of survey-based measures, guidance on evidence gathering (against relevant elements of the Framework) is provided to encourage the use of interviews and focus groups, short ‘sound bites’ taken at activities, and observations. The measurement guidance included in the Toolkit is general in nature, with direction provided to tailor items to an organisation’s project, health focus, Framework coverage, and population(s). This guidance also includes options for organisations able to collect data at multiple timepoints, and organisations that can only collect data at a single timepoint. Specific case studies and template forms are provided within the Toolkit to support its ease of use. Healthway provide guidance and support for use of the Framework and Toolkit to its funded organisations. The Measurement Toolkit is available in Supplementary material.

## 3 Application of the framework

The Healthway Evaluation Framework has to date been (and is being) implemented by State Sporting Associations and peak bodies for the evaluation of health promotion initiatives—targeted at key health areas including mental health and healthy eating—reaching over 150,000 people in Western Australia. Additionally, other community and non-sport organisations have adopted and implemented the Framework to evaluate their health promotion initiatives, including programs targeted at social connection, child and adolescent health, and support for people with disabilities. The Framework has been used to inform project design and objective setting, program logic development, evaluation planning, data collection, and reporting and dissemination for organisations delivering health promotion initiatives. Importantly, the Healthway Evaluation Framework is intended to be adaptable to context—including the intended target outcome or phenomena, the intervention and how it is received, and the social circumstances of the evaluation itself [e.g., power relations; (29)]. As the Framework is designed to be used pragmatically, not all elements are relevant or required for all initiatives. Instead, organisations are encouraged to consider all elements and select Framework elements most pertinent to their project, giving them a scaffold for robust evaluation. The Healthway Measurement Toolkit (see Supplementary material) is designed to support decisions on how to capture each element of the Framework.

To illustrate the use of the Framework for evaluation of a health promotion project—consider an organisations delivering an initiative to provide sport organisations with training and resources to drive positive change related to mental health literacy, support, and environments. The organisations selected the relevant elements of the Framework (10 of the 13 Framework elements), and mapped their plan for data collection (and subsequently their evaluation reporting) to these elements. For instance, they tracked website visits to assess Participation and Engagement, used open-ended text responses to gather insights on Implementation, Adoption, and Fidelity, and collected staff perceptions on changes to their organisations’ ability to deliver effective mental health promotion initiatives to assess Health Promotion Capacity. In their final project evaluation report, they provided in-depth evidence mapped closely to the Framework—an example of how this was reported is included below:

As a result of the Initiative as a whole, the majority of respondents reported improved confidence in communicating with people about mental health topics (77% agree / strongly agree), and in their ability to support others’ mental health and wellbeing (74% agree / strongly agree). Participants felt that their confidence recognizing and responding to mental health issues (78% agree / strongly agree) and critical mental health incidents (87% agree / strongly agree) also improved. 83% of participants agreed or strongly agreed that the resources provided practical strategies to help support others’ mental health and wellbeing. The resources improved participants’ awareness of the mental health and wellbeing support and resources available from [the organisations] (87% agree / strongly agree) and services in the community (83% agree / strongly agree).

From a health promotion funder’s perspective (e.g., in this case, Healthway), the Framework serves to drive consistency in project objectives, project applications, evaluation plans, and evaluation activity across their funding portfolio and their jurisdiction. Greater consistency in evaluation planning and reporting for each project augments Healthway’s capacity for aggregation and monitoring of health impact across their funded organisations, contributing to the health promotion evidence base and enabling clear aggregated signposting and reporting regarding their collective impact. As such, a Framework of this nature can be used by funders to provide a common and compelling language for their own reporting—allowing the organisations to demonstrate accountability and to track progress against strategic plan or organisational vision statements. And, with its focus on qualitative and quantitative data, a Framework of this nature supports a strategic approach to narratives, case studies, and impact ‘storytelling’ embedded within reporting. Beyond these evaluation and reporting functions, data collected in line with the Framework may also provide valuable opportunities for research (when research ethics committee approvals are in place), including subgroup analyses, data aggregation, and examination of contextual factors influencing health promotion outcomes. It is our (and Healthway’s) hope that funding and policy-setting organisations beyond Healthway may find value in using and/or modifying this Framework, and we encourage interested readers to read the Supplementary materials for additional information on the Toolkit, and to support implementation efforts. For instance, we anticipate that the Framework may carry value beyond Western Australia through connection to the work of national organisations such as the *Australian Health Promotion Association*, and beyond Australia’s borders through international networks including the *International Network of Health Promotion Foundations* (www.inhpf.org; of which Healthway is a member).

## 4 Conclusion

The *Healthway Evaluation Framework* addresses key gaps in existing evaluation frameworks relating to practicality, adaptability, and comprehensiveness—it brings together academic rigor, a practical focus, and a consideration of local context. By integrating insights drawn directly from stakeholders with core components of the Ottawa Charter for Health Promotion and established evaluation and implementation science literature, the Evaluation Framework and accompanying Measurement Toolkit provides specific guidance that not only support organisations in demonstrating their impact, but also to build internal capacity to conduct meaningful and rigorous evaluation, and inform strategic decision making. For funders and policymakers, adoption of the Framework across its portfolio and jurisdiction provides a foundation for consistent reporting, showcasing the cumulative impact and strengthening the health promotion evidence base. High-quality evaluation is and will remain paramount within health promotion initiatives—the *Healthway Evaluation Framework* represents a practical, evidence-based, and transferrable resource for documenting and advancing impact in health promotion.
