# Supplementary material for: Measuring delivery and impact in community-based health promotion initiatives: development and overview of the Healthway Evaluation Framework
Source: Front Public Health. 2025 Dec 2;13:1676965. doi: 10.3389/fpubh.2025.1676965 (PMC12705574; doi:10.3389/fpubh.2025.1676965)
Supplement: Supplementary file 1 [file Data_Sheet_1.pdf]

GOVERNMENT OF WESTERN AUSTRALIA

healthway

Healthway Evaluation Framework

Overview & Principles

SEPTEMBER | 2024

Acknowledging Country

This document was prepared by health promotion researchers at The University of Western Australia (UWA) in collaboration with colleagues at Healthway. We wish to begin this document with an acknowledgement that we are extremely fortunate to live and do our work on Noongar Whadjuk boodjar—the land of the Whadjuk people of the proud Noongar nation. We are also mindful that UWA's campus is situated on sacred and beautiful land—between the Derbarl Yerrigan (Swan River), Boorloo (Perth), and Kaarta Gar-up (Kings Park). Whenever we walk our campus, and whenever we travel out across our breath-taking State, we are grateful for the reminder of the truly special country upon which we live, play, and do our work.

We pay our deepest respects, and offer our thanks, to Noongar Elders past, present, and emerging. We value their wisdom and their guidance in the work that Healthway does to connect and improve the lives of Western Australians. We extend that respect to all Aboriginal and Torres Strait Islander peoples.

The purpose of Healthway's Evaluation Framework

Healthway's Evaluation Framework presents the priorities and best practices for evaluating the impact of a funded project.

It is designed to capture impact that is broader than message awareness and project reach, and empower organisations to capture the full range of project outcomes.

Healthway will provide guidance and support around using the Toolkit and establishing appropriate measures for your project.

The idea is, through using the Framework and the accompanying Toolkit, Healthway will assist you to find efficient and effective ways to measure project outcomes.

Why is ‘Evaluation and Measurement of Process and Impact’ important?

Healthway’s Evaluation Framework plays an important role in guiding our investments to support health and wellbeing outcomes in the Western Australian (WA) community.

It also allows a consistent focus on measuring the impact of funded projects as we deliver our new strategic plan *Creating a healthier WA together 2024-2029.*

Healthway will use the Framework to:

- allocate resources based on demonstrated impact and effectiveness of different programs,
- enhance transparency in decision making and communication with our partners,
- assess effectiveness,
- ensure accountability, and
- support ongoing learning.

Ultimately it will enable Healthway to demonstrate the impact of its investment to achieve its vision of a healthier WA.

Overview - A Framework of Evaluation Measures For Healthy Partnerships / Communities

SETTING INDIVIDUAL PROGRAM OBJECTIVES

ACTIVITY

- Participation and engagement
- Promotional and educational activities
- Implementation, adoption, fidelity

KNOWLEDGE, ATTITUDE, BEHAVIOUR

- Health knowledge and attitudes
- Health behaviour
- Social capital and social health

ORGANISATIONAL

- Organisation reach and mission
- Health promotion capacity
- Volunteering

SUSTAINABILITY

- Maintenance and capacity
- Policy
- Partnerships
- Structural reforms

Process Evaluation (Activity)

Impact Evaluation (Knowledge, Attitude, Behaviour; Organisational; Sustainability)

EVALUATING INDIVIDUAL PROGRAM OBJECTIVES

Why it's important to measure Individual project objectives
(and even more important to think about in planning your project):

Before you begin, take this step to reflect on project success and outcomes, to encourage at application stage that project objectives are mapped as closely as possible to the Pillars in the Framework (e.g., Activity; Knowledge, Attitude, Behaviour)

- This first step is included to encourage you to first plan and later reflect on your project goals and objectives.
- Healthway will work with you to align the objectives of funded projects with their strategic plan and the pillars of this Evaluation Framework wherever possible.
- Developing a program logic will assist with identifying project objectives. Healthway can provide more information to assist you with this process.
- Aligned project objectives help to strengthen the collective impact of funded projects for several key reasons:
    - Clarity of purpose - aligned program objectives provide a clear understanding of the overarching goals and outcomes that Healthway is working towards with all funded groups.
    - Synergy and co-ordination – allows opportunities for collaboration e.g. sharing knowledge, pooling resources, and co-ordinating efforts more effectively to achieve common outcomes.
    - Shared vision – creates a cohesive narrative that can be shared with the community and can inspire community engagement and participation.
- With aligned project objectives, Healthway will be able to undertake a more accurate assessment of collective outcomes.
- Please refer to the Toolkit for ideas about assessment and actions within this element.

ACTIVITY

Participation and engagement

Education and awareness raising activities

Implementation, adoption, fidelity

What does this element of the framework mean?

The number and type of people / groups that are participating in a funded activity - including those within the recipient organisation, those delivering activities on the ground, and the recipients or consumers of those activities.

Why it's important to measure **Participation and Engagement:**

- It's important to consider the participation and engagement from all possible groups associated with your program or activity. These groups may include:
  - Representatives from the funded organisation.
  - Volunteers, coaches, educators, instructors, health promotion officers.
  - Recipients or consumers of the health promotion initiative.
- Quantifying participation can help you reflect on your programs/initiatives, and can also help you understand who took part and why.
- Measuring participation against Healthway's and/or your organisation's key target groups allows us to see if the activity is not just 'reaching people', but is reaching the 'right' people (e.g. regional, culturally and linguistically diverse (CaLD), Aboriginal, children and young people, etc).
- The data each funded organisation collects will be collated by Healthway - so Healthway can examine which populations are being supported and which population groups are being missed.
- Please refer to the Toolkit for measurement options.

ACTIVITY

Participation and engagement

Education and awareness raising activities

Implementation, adoption, fidelity

What does this element of the framework mean?

A comprehensive record of the various activities, events, strategies, and initiatives provided within the funded project.

Why it's important to measure
Education and awareness raising activities:

- Allows us to capture all activities, events, and initiatives that were delivered within the funded project.
- This includes any activities aimed at blocking undesirable brands or messages.
- Within any activity there will be many 'layers' to measure.
    - For example, your organisation may have delivered an event (a measurable activity in itself) that included several discrete strategies or initiatives focused on project objectives (e.g. providing Act Belong Commit signage, delivering an educational workshop, preparing and distributing take-home booklets and resources).
    - In this instance, comprehensive reporting would capture all of these elements.
- From time to time Healthway may undertake field surveys, which gather additional data such as measuring participant's awareness of different initiatives and strategies at funded events.
- Please refer to the Toolkit for measurement options.

ACTIVITY

Participation and engagement

Education and awareness raising activities

Implementation, adoption, fidelity

What does this element of the framework mean?

All remaining 'delivery' considerations that inform if the project / activities were implemented as planned and if they were accepted by the target group.

Why it's important to measure Implementation, adoption, fidelity:

- This looks at all other 'ingredients' involved in undertaking the funded program.
  - **Implementation** examines if the funded program/activity was delivered as intended and if not, why there were variations in implementation and the impact they had on the activity.
  - **Adoption** measures if the funded program/activity was received by participants as intended and if not, why there were variations in adoption and the impact they had on the activity.
  - **Fidelity** looks at whether all the promised activity components were delivered as planned in the way they were planned and if not, why there were variations and what impact they had on the activity.
- This element is really looking at how appropriate or accepted the program/activity was for the target group.
- Please refer to the Toolkit for measurement options.

KNOWLEDGE, ATTITUDE, BEHAVIOUR

Health knowledge and attitudes

Health behaviours

Social capital and social health

What does this element of the framework mean?

Knowledge / understanding and motivation to access, understand, appraise, and apply health information—to support appropriate judgments and decisions concerning health, disease prevention, and health promotion.

Why it's important to measure Health knowledge and attitudes:

- Health knowledge and attitudes (also known as health literacy) reflects people's
    - ability and motivation to find, understand, and use health information,
    - their attitude towards health, and
    - their confidence to take appropriate action to improve their health.
- This element measures awareness, understanding and intentions to change behaviour around the health messages Healthway promotes (e.g. LiveLighter®, Think. Mental Health, Fuel to Go & Play), as well as knowledge, understanding and motivation regarding specific health behaviours (e.g. increasing physical activity).
- For some projects and activities, there will be a need for age-appropriateness considerations and culturally sensitive / appropriate considerations.
- If we can collect information both before and after a project or activity, this is an area where programs may be able to show change through people participating in funded projects / activities.
- Please refer to the Toolkit for measurement options.

KNOWLEDGE, ATTITUDE, BEHAVIOUR

Health knowledge and attitudes

Health behaviours

Social capital and social health

What does this element of the framework mean?

Changes in health behaviours that are relevant to project aims - includes physical and mental health (and wellbeing) indicators.

Why it's important to measure Health behaviours:

- Health behaviours are health-related practices such as exercise, that can improve or damage the health of individuals or community members. We are looking to measure any change in physical and/or mental health behaviours.
- Health behaviours may include:
  - Physical activity engagement (and linked outcomes—sports club, fitness centre attendance, participation in informal sport).
  - Dietary intake (e.g., sugary drinks, fruit and vegetables, junk food).
  - Alcohol (or other drug) intake.
  - Smoking (including e-cig / vaping).
  - Indications of high wellbeing - feeling good and functioning well, practicing behaviours to protect mental health.
- If required, Healthway will work with you to determine the best way to measure a change in health behaviour.
- Please refer to the Toolkit for measurement options.

KNOWLEDGE, ATTITUDE, BEHAVIOUR

Health knowledge and attitudes

Health behaviours

Social capital and social health

What does this element of the framework mean?

The social norms, networks, cohesion, trust, and/or supports that underpin cooperation, provide resources and development opportunities, and add value to an individual's experience.

Why it's important to measure Social capital and social health:

- Social capital refers to the networks and social connections within a community that enable co-operation and mutual support, while social health relates to an individual's overall wellbeing within the context of their social relationships.
- Measurement of social capital and social health is of interest for Healthway, yet is challenging due to their subjectivity, complexity and cultural variability.
- Measurement of social capital and social health would only be undertaken at the request of, and in conjunction with Healthway.
- Please refer to the Toolkit for measurement options.

ORGANISATIONAL

Organisation reach and mission

Health promotion capacity

Volunteering

What does this element of the framework mean?

Whether and how the funded program/project supported your organisation's reach and mission; and, in a secondary sense, Healthway's reach and mission.

Why it's important to measure Organisation reach and mission:

- These measures examine how well your Healthway funded projects align with your own organisational purpose.
- **Reach** is a measure of participation. It looks at the benefits (or challenges) to your organisation associated with the project or activity in terms of its reach—both generally and into specific sub-populations.
    - For example, did the project expand your organisation's reach in terms of specified target areas / populations?
    - Were there any implications of the activity in terms of limiting reach into other specific sub-populations? (e.g. perhaps through the funded activity the organisation reached more Aboriginal communities than planned, but did this come at the expense of reach into CaLD communities?).
- Ideally, this information is aligned to Healthway priority population groups.
- **Mission** is an explanation of your organisation's reason for existence. Assessing this element may reflect whether the project or activity advanced or hindered your organisation's mission in a general sense and also involves some reflection on whether and how the project advanced or contributed to Healthway's mission.
- Please refer to the Toolkit for measurement options.

ORGANISATIONAL

Organisation reach and mission

Health promotion capacity

Volunteering

What does this element of the framework mean?

The short, medium, and longer-term change to your organisation's capacity for designing and delivering health promotion and evidence-building activities.

Why it's important to measure Health promotion capacity:

- One intended outcome of Healthway funding is an increase (ideally a lasting increase) in the health promotion capacity of funded organisations.
- Health promotion capacity reflects the potential medium to longer-term benefits of investing in organisations to deliver health promotion programs and robust evaluation.
- That capacity may include, for example:
  - Adding to human capacity (e.g. new staff / volunteers, FTE).
  - Improving human capacity (e.g. adding to staff or leader knowledge, skill, confidence, health literacy).
  - Health promoting activities included in strategic planning documents and/or implementation plans.
  - Health promoting activities included in annual budgets.
  - Other changes to policy or practice (or intentions to do so).
  - Improvements in health promotion evaluation planning and activities.
  - Perception of health promotion support or supportive environments.
- Ideally evaluation of this element should clearly indicate in which area/s the expertise or capacity or legacy exists.
- Please refer to the Toolkit for ideas about assessment and actions within this element.

ORGANISATIONAL

Organisation reach and mission

Health promotion capacity

Volunteering

What does this element of the framework mean?

Assessing all benefits and costs to your organisation regarding volunteers (and to volunteers themselves).

Why it's important to measure Volunteering:

- Where possible and relevant, this element focuses specifically on the project impact in terms of volunteers and volunteer capacity.
- Any further consideration for volunteer-relevant data captured in this element may include measuring (amongst others):
  - The time and commitment required, which could be translated into a cost per activity or a cost per project basis—needed to adequately 'staff' a project with volunteers (i.e. volunteer 'costs').
  - The costs directly to volunteers themselves (e.g. stress, time, compassion burden).
  - Other organisational benefits gained from volunteers.
  - The benefits gained by the volunteers themselves.
- This would allow for Healthway and other interested parties to determine the hidden volunteer-related costs and risks associated with projects.
- Please refer to the Toolkit for measurement options.

SUSTAINABILITY

Maintenance and capacity

Policy change

Partnerships

Structural reforms

What does this element of the framework mean?

An organisation's overall capacity to maintain the funded project or initiative beyond its initial funding window.

Why it's important to measure Maintenance and capacity:

- The 'Maintenance' element will assess considerations / challenges like:
  - What will happen to the project / initiative in the longer-term (and why)?
  - Will modifications be needed?
  - What will support maintenance of those activities? And what will support continued effectiveness of the activities for recipients / consumers?
  - Can organisations (and other partners) sustain the project following the funding period (and the staff, infrastructure, etc to do so)?
  - Can evaluation and impact monitoring be sustained around project continuation?
  - Is ongoing funding in place (e.g. organisation's budget)?
- The notion of 'Capacity' here is focused on capacity to enable program or project maintenance (rather than health promotion or other aspects of organisational capacity).
- Please refer to the Toolkit for measurement options.

What does this element of the framework mean?

Development, implementation, and sustainability of any (internal or external) policy change resulting directly or indirectly (e.g. through a change in organisational capacity) from the funded project.

Why it's important to measure Policy:

- Policy change is an important outcome to evaluate as it can have a strong impact on community health behaviour and norms.
- Policy change may occur within your organisation or with external partners (e.g. multiple organisations, within peak / governing body, Government).
- Emphasis here is on any and all policy change (including the creation of a policy) that results directly or indirectly from the funded project:
  - Direct: The funded activity results in some policy change within or beyond the organisation.
  - Indirect (example): The funded activity results in a change to organisation's health promotion capacity, and this increase in capacity may in turn drive policy change.
- Healthway can use this information to bring greater consistency to the policies that organisations look to implement which will result in greater impact across the community.
  - Organisations seeking funding will be guided by Healthway during the development of their request to consider and include their intended or aspirational policy objectives (e.g. a healthy cooking program built into organisation's core business), and/or their advocacy efforts for higher-level policy change (e.g. at Government or peak body level)
- It is also important to measure the implementation and sustainability aspects of any policies.
- Please refer to the Toolkit for measurement ideas.

SUSTAINABILITY

Maintenance and capacity

Policy

Partnerships

Structural reforms

What does this element of the framework mean?

The quality and nature of partnerships with external organisations that are necessary to support program sustainability and (if applicable) scalability.

Why it's important to measure Partnerships:

- This considers whether and how the organisation and the specific project or initiative has the necessary partners to deliver and sustain the activity. This may include considerations such as:
  - Is there strategic and practical suitability of partners?
  - Are partnerships sufficiently strong / robust?
  - Is there demonstrated commitment from partner organisations (e.g. government, industry, hospital / clinical, non-profit, health promotion, etc).
- The aim here is to determine the nature of the partnership and measure partnership outcomes based upon the nature of the relationship. This will help Healthway to assess areas in which there are partnership needs or 'gaps'.
- Nature of the partner relationship should be clarified and may include contributions such as:
  - Funding partner, hosting of project platforms / online materials, promotion and/or access to and facilitating interactions with populations (e.g. Aboriginal, remote, refugee, etc), program delivery aspects, advocacy with peak bodies or Government, evaluation and quality improvement.
- Different types of Healthway's partner organisations are shown on the next slide.
- Please refer to the Toolkit for measurement ideas.

CLASSIFICATION OF HEALTHWAY PARTNERSHIPS

	STRATEGICALLY ALIGNED	MUTUALLY BENEFICIAL	ALLIANCE
	Partners whose goals are aligned with Healthway	Partners whose goals may not be aligned but who can provide a mutual benefit	Partners who view Healthway's goals as aligned with their own and can provide added value to Healthway funding
PARTNERSHIP OPPORTUNITIES*	• Health related non-government organisations e.g. Cancer Council WA<br>• Academic Institutions<br>• Research bodies<br>• Public Health Units<br>• Department of Health<br>• Department of Local Government, Sport and Cultural Industries<br>• Mental Health Commission	• Sporting associations<br>• Arts organisations<br>• Racing organisations<br>• Community groups<br>• Local sporting clubs<br>• Local governments<br>• Schools<br>• Recreation centres	• Lotterywest<br>• WA Local Government Association<br>• Health insurance companies<br>• Philanthropic companies<br>• Private Foundations

* The list of partners presented is for illustration purposes. It is not exhaustive.

SUSTAINABILITY

Maintenance and capacity

Policy

Partnerships

Structural reforms

What does this element of the framework mean?

Physical, structural, and organisational changes made within the organisation (or the organisation's reach) that contribute to the creation of healthy environments and project sustainability.

Why it's important to measure Structural reforms:

- Structural reform is closely linked to policy change – as it often reflects the strategies implemented when a policy is introduced.
- Structural reform can include both physical and/or organisational structural reforms
    - Physical reforms lead to the creation of healthy environments, examples include the provision of alcohol-free areas, smoke-free venues, healthy food offerings.
    - Organisational reform may include the funded organisation creating new roles, redistributing workloads, creating new working groups or committees, and/or restructuring their organisational chart/s to enable project continuation.
- Potential structural reforms can be identified at the time of request to ensure alignment with Healthway's objectives, and that appropriate strategies are included as part of the request.
- Data should be collected on the number of changes made across the entire program as well as estimates of the impact on the number of people exposed to the structural change.
    - For example, "5000 participants were presented with an increased number of healthy food options; subsequent behaviour or attitudinal changes included…"
- Please refer to the Toolkit for measurement options.

Alignment with Healthway Logic Model

Mapping the new Evaluation Framework against Healthway's current Logic Model

Figure below demonstrates how proposed evaluation pillars map onto all logic model components (see 'key')

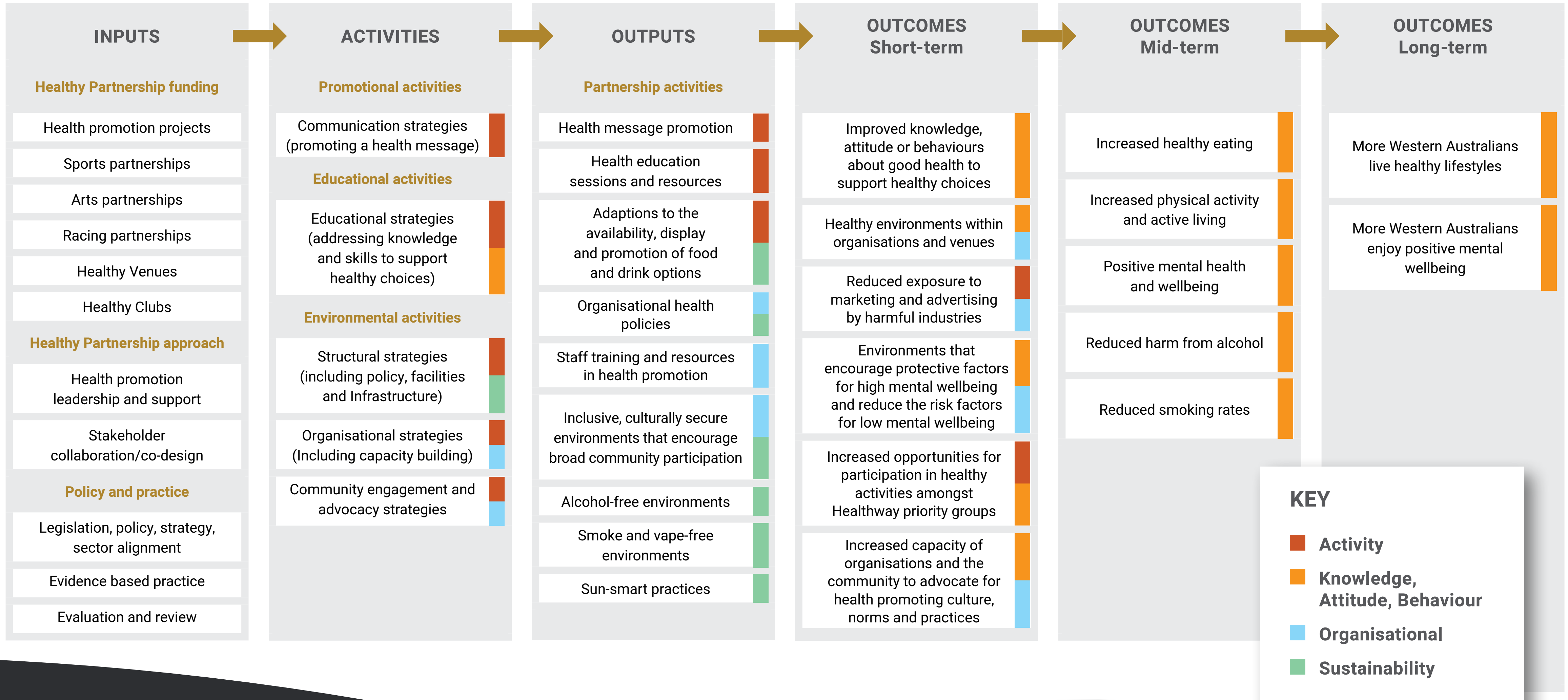

GOVERNMENT OF WESTERN AUSTRALIA

healthway

Street Address:
Level 2, 38 Station Street
Subiaco WA 6008

Postal Address:
Locked Bag 66
Subiaco, WA 6904

Telephone: 133 777
Email: healthway@healthway.wa.gov.au
Media enquiries email: media@healthway.wa.gov.au

Healthway. (2024). Healthway Evaluation Framework: Overview & Principles. Government of Western Australia.

©Healthway 2024
This work is copyright. It may be reproduced in whole or in part for study or training purposes subject to the inclusion of an acknowledgement of the source. It may not be reproduced for commercial usage or sale. Reproduction for purposes other than those indicated above requires written permission from Healthway.
