# Supplementary material for: Measuring delivery and impact in community-based health promotion initiatives: development and overview of the Healthway Evaluation Framework
Source: Front Public Health. 2025 Dec 2;13:1676965. doi: 10.3389/fpubh.2025.1676965 (PMC12705574; doi:10.3389/fpubh.2025.1676965)
Supplement: Supplementary file 2 [file Data_Sheet_2.docx]

**Supplementary Material S2**

To view the Healthway Measurement Toolkit on the Open Science Framework platform, please visit: <https://osf.io/jwkvt/overview?view_only=9250bb2cdbd54a2a898a251635861913>

The Framework and Toolkit can also be accessed on the Healthway website ([www.healthway.wa.gov.au](http://www.healthway.wa.gov.au)).

*Please note:* This Toolkit is provided as a guide for how elements of the Healthway Evaluation Framework can be measured and implemented. The Toolkit was originally designed for use by organisations funded by Healthway. Consequently, throughout the Toolkit, there are references to support provided by Healthway to assist organisations in the development and delivery of evaluation measurement items. This is only relevant to Healthway-funded organisations, and not broader readers of this manuscript and the Toolkit. Any changes to the Toolkit will be described in the Open Science Framework project linked above.
